# Differences in motor unit firing properties of the vastus lateralis muscle during postural and voluntary tasks

**DOI:** 10.3389/fphys.2022.955912

**Published:** 2022-09-30

**Authors:** Toshiyuki Aoyama, Yutaka Kohno

**Affiliations:** ^1^ Department of Physical Therapy, Ibaraki Prefectural University of Health Sciences, Ibaraki, Japan; ^2^ Centre for Medical Sciences, Ibaraki Prefectural University of Health Sciences, Ibaraki, Japan

**Keywords:** motor unit, recruitment, postural task, voluntary task, vastus lateralis

## Abstract

The firing properties of the motor units are usually affected by the motor task. However, it has not been clarified whether the firing properties of the motor units of a specific muscle are different between postural and voluntary tasks. Therefore, this study investigated whether the recruitment and rate coding of the motor units differ between these two motor tasks. Thirteen healthy volunteers performed trapezoidal muscle contraction with a target value of 15% maximum electromyography (EMG) activity by voluntary left knee extension in the sitting position (voluntary task) and postural maintenance in the semi-squatting position (postural task) with a knee flexion angle of 30°. We obtained four channels of surface EMG activity during each task from left vastus lateralis muscle. We extracted the firing properties of individual motor units using the EMG decomposition algorithm. The recruitment threshold and motor unit action potential amplitude were significantly lower in the postural task than in the voluntary task, and conversely, the mean firing rate was significantly higher. These results were explained by the preferential recruitment of motor units with higher recruitment threshold and amplitude in the voluntary task, while motor units with lower recruitment threshold and higher firing rate were preferentially recruited in the postural task. Preferential activation of fatigue-resistant motor units in the postural task is a reasonable strategy as it allows for sustained postural maintenance. We provide the first evidence that motor unit firing properties are clearly different between postural and voluntary tasks, even at the same muscle activity level.

## 1 Introduction

The tension produced by muscles depends largely on the recruitment and rate coding of the motor units that comprise the spinal motoneurons and muscle fibers they innervate. The order in which motor units are recruited essentially follows Henneman’s size principle (Henneman, 1957). According to this principle, small-cell motoneurons, which are characterized by a low threshold and innervate high fatigue-resistant muscle fibers, are recruited initially, whereas the large-cell motoneurons are gradually recruited as the demand for force increases. This principle allows precise execution of the motor behavior at low force levels as well as contributes to the preservation of the fast motor units that are less resistant to fatigue to allow the execution of high force-level motor tasks (Hodson-Tole and Wakeling, 2009). However, some factors other than the size principle affect the recruitment and rate coding of motor units. In this context, previous studies have shown that the motor unit firing properties change depending on the biomechanical factors, such as muscle length (Kennedy and Cresswell, 2001; Hudson et al., 2017) and motor tasks, including the direction (Desnedt and Gidaux, 1981; ter Haar Romeny et al., 1982; Thomas et al., 1986; van Zuylen et al., 1988) or velocity of movement (Oliveira and Negro, 2021), and type of muscle contraction (isometric or dynamic contraction) (Thomas et al., 1987). Therefore, although the firing properties of motor units generally follow the size principle, they are rationally controlled by the mechanical properties of the muscles and the demands of the motor task.

There are two contrasting types of human motor tasks, namely postural and voluntary tasks, which display several neurophysiological differences. A previous study revealed that the motor evoked potential amplitude and latency induced by transcranial magnetic stimulation (TMS) differ between these two motor tasks (Ackermann et al., 1991). In addition, Guzmán-López et al. (2015) revealed that the modulation of the H-reflex amplitude induced by TMS differed between these tasks. Therefore, voluntary and postural muscle contractions are presumably mediated by different neurophysiological mechanisms. It has long been established through several evidence-based studies that the properties of motor units differ depending on the role required of the individual muscles, such as postural maintenance and voluntary movements (Belanger and McComas, 1981; Bellemare et al., 1983). For example, the soleus muscle, which is a postural muscle, predominantly innervates fatigue-resistant muscle fibers (Burke et al., 1974). This is reasonable because postural muscles, which require sustained muscle contraction, must have a high resistance to fatigue. In contrast, the lateral gastrocnemius muscle, which is less associated with postural maintenance than the soleus muscle (Heroux et al., 2014), was revealed to innervate considerably lower percentage of fatigue-resistant muscle fibers than the soleus muscle (Burke and Tsairis, 1973; Gillespie et al., 1987). Therefore, individual muscles are likely to have an optimized composition of motor units for their required physiological role. However, it is not fully understood whether the behavior of motor units in a specific muscle is affected by the two motor tasks, postural and voluntary motor tasks. A previous study reported that the mean firing rate of soleus motor units was different between postural and voluntary tasks (Mochizuki et al., 2006). However, this study is not suitable for comparing the characteristics of the motor units between postural and voluntary tasks because the level of muscle activity was not equally the same in the two motor tasks. Therefore, the present study aimed to clarify the differences in the recruitment and rate coding of the motor units of the vastus lateralis muscle between postural and voluntary tasks, while matching the muscle activity levels of both tasks. Additionally, we clarified whether the difference in the behavior of the motor units between the two motor tasks was due to the recruitment of distinct motor units with different firing properties or to the changes in the firing properties of the motor units common to both tasks. These clarifications would provide a framework for the rational control of the two motor tasks in the central nervous system and facilitate the application of these two motor tasks in muscle strength training. In general, since sustained muscle activity is required to maintain a standing posture, we hypothesized that low-threshold motor units, which are considered more resistant to fatigue, would be preferentially recruited in the postural task than in the voluntary task.

## 2 Methods

### 2.1 Participants and ethical approval

Thirteen healthy volunteers (9 men and 4 women) with a mean (SD) age of 21.9 (4.5) years participated in this study; two of them were excluded because they were unable to produce the target strength of muscle activity (15% maximum voluntary contraction [MVC]) in the postural task, as described below. All participants had no history of neurological or orthopedic disorders. All of the participants provided written informed consent prior to participation in accordance with the Declaration of Helsinki, and study protocols were approved by the ethics committee of the Ibaraki Prefectural University of Health Sciences (approval number: 877).

### 2.2 Electromyography

**FIGURE 1:**
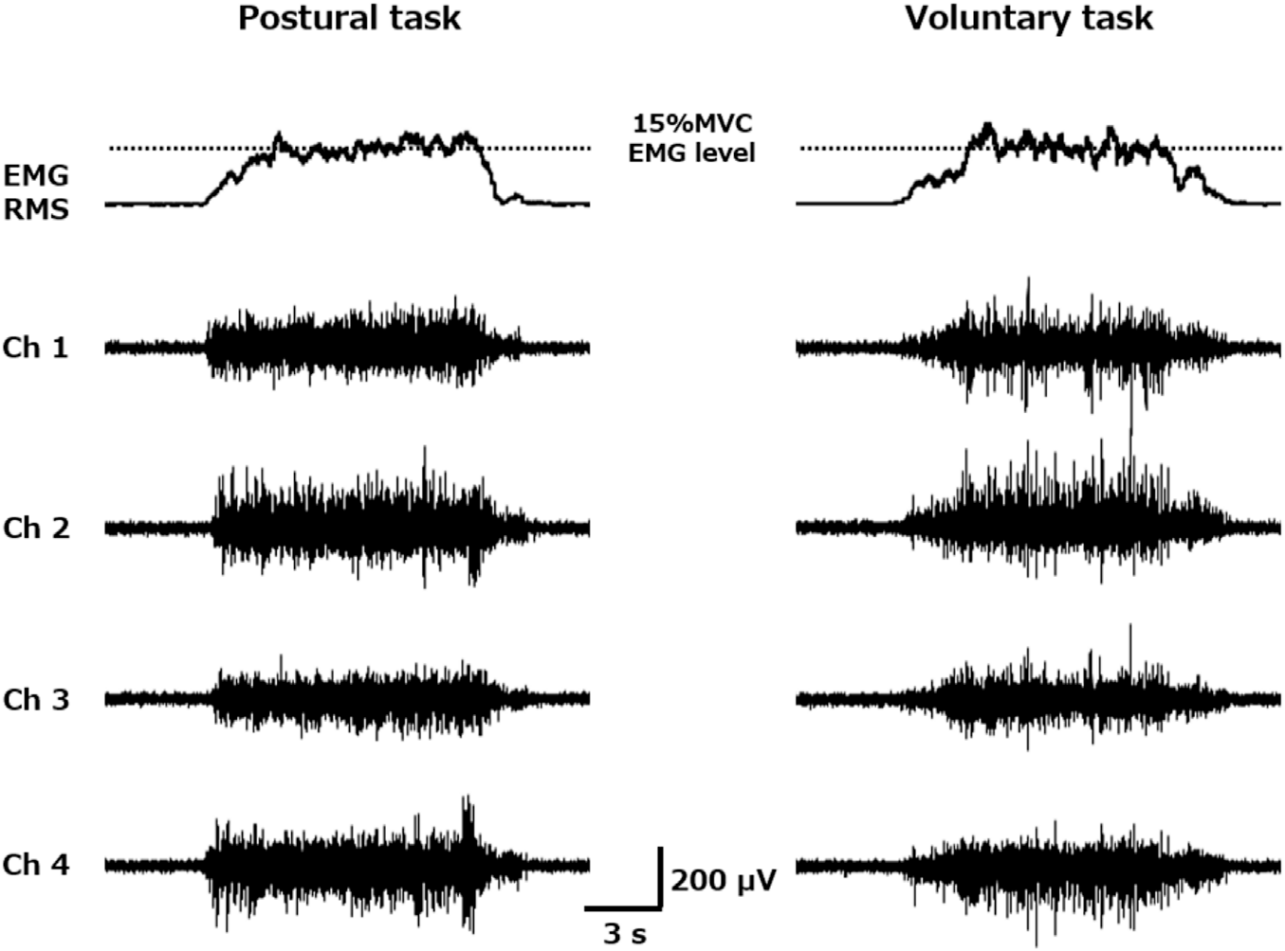
Root mean square (RMS) envelope of the electromyography (EMG) activity and four channels of surface EMG raw waveforms obtained during trapezoid muscle contraction. Each participant performed trapezoidal muscle contractions (2.5 s for the ramp-up phase, 8 s for the plateau phase, and 2.5 s for the ramp-down phase) with a target value of 15% maximum voluntary contraction EMG level during both postural (left) and voluntary (right) tasks. The motor unit firing properties were extracted from each of the four channels of the surface muscle activity obtained from the vastus lateralis muscle*.*

Before attaching the electromyography (EMG) electrodes, the participant’s skin was wiped with alcohol and rubbed with an abrasive skin preparation gel. EMG electrode comprising four pins arranged in a diamond shape at 5-mm intervals was placed on the left vastus lateralis muscle and positioned 2/3 on the line from the superior anterior iliac spine to the lateral side of the patella (Hermens et al., 1999). A reference electrode was placed on the lateral epicondyle of the femur. Four channels of muscle activity were recorded from these four pin electrodes (Figure 1). EMG signals were amplified at an increase of 1,000 using a Trigno wireless system (Delsys Inc., United States) and bandpass filtered at 20 Hz–450 Hz. The sampling rate was set at 2,222 Hz and EMG signals were stored on a laboratory computer for subsequent analysis.

### 2.3 Measurement of the MVC

At the start of the experiment, each participant performed warm up for approximately 10 min, including static and dynamic stretching. A Cybex 6,000 isokinetic dynamometer (Lumex Inc., United States) was used to measure MVC. The participants were seated with the knee at 30° flexion. Before MVC measurement, isometric knee joint extension was performed at 50%, 70%, and 90% of subjective maximum muscle contraction for 5 s each. After a 3-min rest, MVC was measured twice for 4 s in 30° flexion of the knee joint. During the MVC measurements, the researcher (physical therapist) verbally encouraged the participants to exert maximum muscle strength. A 3-min break was allowed between each MVC measurement. The same MVC measurements were taken after all motor tasks were performed to ensure that the motor tasks described below did not cause muscle fatigue.

### 2.4 Motor tasks

**FIGURE 2:**
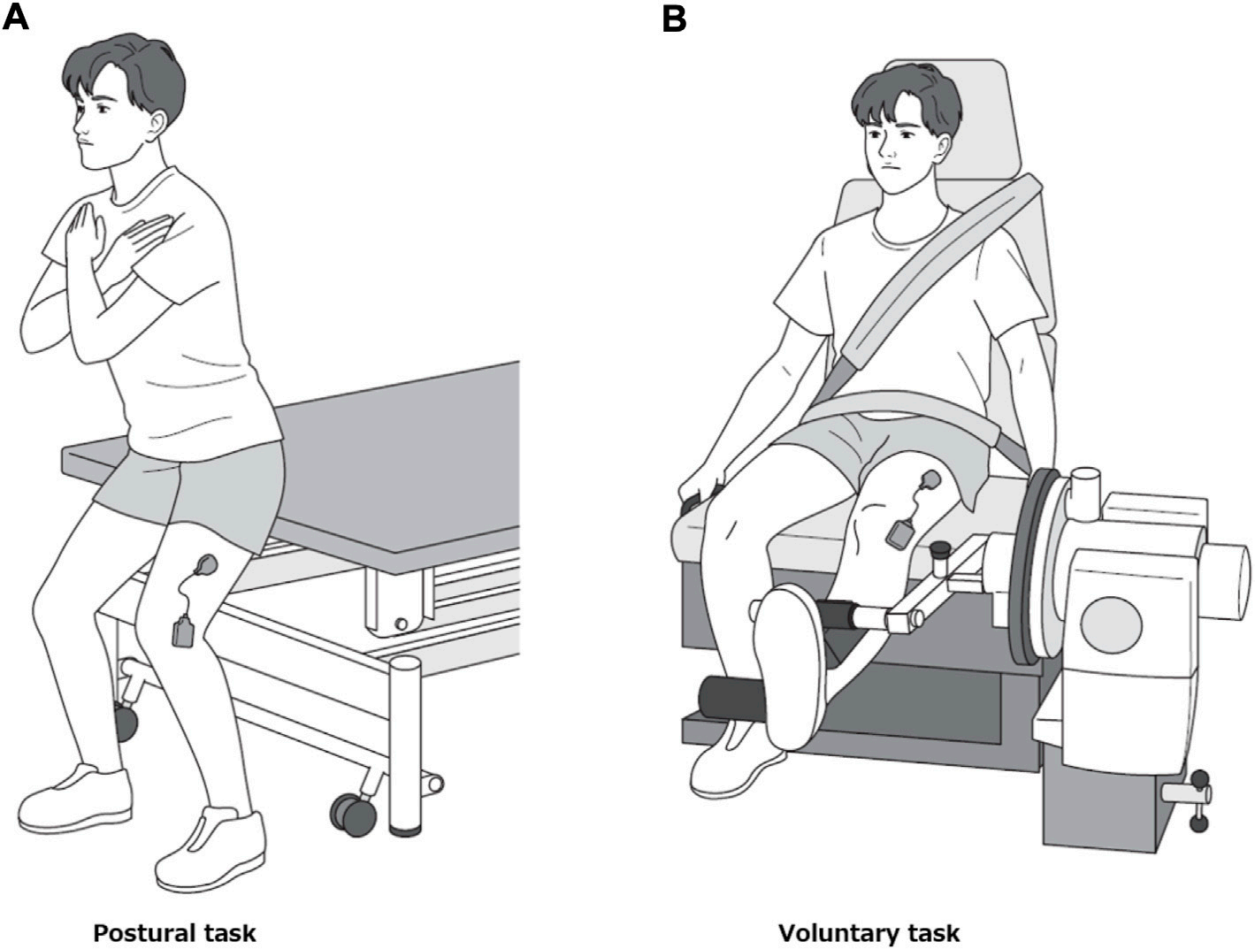
Motor tasks. Representative picture of a participant performing the postural task **(A)** and the voluntary task **(B)**.

Each participant performed two motor tasks, postural and voluntary. During the first task, the participants stood in a semi-squatting position with the knee bent at 30° flexion (Figure 2A). They performed trapezoidal contraction by adjusting the amount of body weight applied to the left lower extremity while maintaining the same posture. The postural task was performed with the buttocks in contact with the edge of the treatment table whose height was adjusted to match the position of their buttocks to prevent the knee flexion angle from changing. The voluntary task was performed by isometric trapezoidal contraction of the knee extensors at 30° knee flexion using Cybex 6,000 similar to the MVC measurement (Figure 2B). In each of the two tasks, a 27-inch computer monitor was placed in front of the participants at a distance of 1.5 m to provide visual feedback. On this monitor, the target values of muscle activity to perform trapezoidal contraction and EMG root mean square (RMS) values (Westgaard and De Luca, 2001; De Luca et al., 2006) (window length 0.3 s) obtained from the vastus lateralis muscle were projected. For both tasks, the target level was set at 15% MVC-EMG level, ramp-up phase at 2.5 s, plateau phase at 8 s and ramp-down phase at 2.5 s (Figure 1). We did not use a high MVC intensity (30%–80% MVC) as used in previous studies (Boccia et al., 2019; Mota et al., 2020; Reece and Herda, 2021), because we had already confirmed in preliminary examination that some participants had difficulty in producing EMG levels greater than 15% MVC in the postural task. Each motor task was practiced well in advance until the level of muscle activity could match the target level. Each motor task was measured three times, with a 3-min rest between trials, and the order of the two motor tasks was counterbalanced to avoid their effects.

### 2.5 Decomposition of the motor units

The firing trains of the individual motor units were extracted from the four channels of EMG activity (Figure 1), using Neuromap software (Delsys Inc., United States). The accuracy of the decomposition was calculated using the decompose–synthesize–decompose–compare (DSDC) method (Nawab et al., 2010; De Luca and Contessa, 2012; De Luca et al., 2015). The recorded surface EMG signal s (n) was decomposed into each MUAP train. A reconstructed signal y (n) was synthesized by summing-up the MUAP trains and time-varying Gaussian noise. Then, the reconstructed signal y (n) was decomposed again and compared to the previously decomposed MUAP trains. The errors derived from the comparison of motor unit firing times and MUAP shapes between y (n) and s (n) were expressed as false positives (FP) and false negatives (FN).

For all firings of each MU, the accuracy was calculated as follows:

$$Acuuracy=1-\sum \frac{FP+FN}{TP+TN}$$ (1)

where *TP* represents true positives and *TN* represents true negatives. To exclude motor units with low decomposition accuracy from the analysis, only those with accuracy > 80% were included in this analysis based on previously used criteria (Madarshahian et al., 2021; Madarshahian and Latash, 2021). For further analysis, we examined the following four items: 1) number of motor units; 2) motor unit action potential (MUAP) amplitude, calculated as the maximum amplitude of the positive and negative peaks of the MUAP detected from the four EMG channels (Contessa et al., 2016; Contessa et al., 2018); 3) motor unit recruitment threshold, calculated as the EMG level at which the motor unit began to firing; and 4) mean firing rate of the motor unit, calculated from the inverse of the inter-pulse intervals between motor unit firings during the plateau phase of the trapezoid contraction.

### 2.6 Data analysis

Out of the three trials measured in each of the two motor tasks, the ones in which the average EMG activity was closest to the target level of 15% MVC during the plateau phase of the trapezoid contraction was included for the analysis.

All statistical analyses were conducted using SPSS, version 23.0 (IBM, United States). For all statistical comparisons, the significance level was set at α = 0.05. All variables were first tested for normality using the Shapiro–Wilk test. If normality was rejected, a non-parametric test was used; otherwise, a parametric test was used.

#### 2.6.1 Performance of the two motor tasks undertaken

Maximum knee extension torques measured before and after two motor tasks were compared using a paired *t*-test, to compare whether there was a difference in muscle activity exerted during the plateau phase of the two motor tasks. In addition, the EMG activity obtained from the surface electrode is considered to be theoretically lower than the original muscle activity level because of the effect of EMG amplitude cancellation caused by the overlap of the positive and negative phases of the MUAPs (Keenan et al., 2005; Keenan et al., 2006). Therefore, the following procedures were used to simulate actual muscle activity, unaffected by EMG amplitude cancellation. First, all extracted MUAPs were rectified to prevent EMG amplitude cancellation due to the overlap of the active and negative phases of EMG amplitude (Keenan et al., 2005). Next, the simulated EMG area in the plateau phase of trapezoid contraction was calculated by multiplying the area of all rectified MUAPs by their respective number of firings and summing them together. The Wilcoxon signed-rank test was used to determine whether the simulated EMG area differed between the voluntary and postural tasks.

#### 2.6.2 Average data of the motor unit firing properties for each individual participant

The MUAP amplitude, mean firing rate and recruitment threshold were averaged for each individual participant. We examined differences between postural and voluntary tasks in each of these firing properties and the number of motor units using paired *t*-test.

#### 2.6.3 Relationship between recruitment threshold and mean firing rate

A linear regression analysis was conducted to identify the relationship between the recruitment threshold and the mean firing rate of the motor units pooled from all participants in each of the two motor tasks. We further tested the homogeneity of their slopes *via* analysis of covariance.

#### 2.6.4 Firing properties of the motor units recruited in both motor tasks

To clarify whether the difference in the firing properties of the motor units between the two motor tasks was due to the recruitment of motor units with different firing properties or due to the changes in the firing properties of the motor units commonly recruited for both tasks, we identified the motor units commonly recruited during the two tasks. Specifically, for each combination of individual motor units identified in the two motor tasks, we assumed that the motor units were identical if the cross correlation of the shape of MUAP was > 0.8 (Farina et al., 2003; Oliveira and Negro, 2021) and the difference in amplitude was < 40% (Farina et al., 2003) in all four channels. Wilcoxon’s signed-rank sum test was used to examine the differences between the two motor tasks for the recruitment threshold, MUAP amplitude and mean firing rate of the motor units that were commonly recruited in both tasks.

#### 2.6.5 Firing properties of motor units recruited only in one of the motor tasks

Motor units that were not similar were considered to be independently recruited motor units in the respective motor tasks. Mann–Whitney *U* test was performed for the recruitment threshold, MUAP amplitude and mean firing rate of the motor units extracted independently for both tasks.

To further characterize the recruitment of independently extracted motor units, we classified the recruitment threshold, MUAP amplitude and mean firing rate into each of the following three relative groups based on their respective distributions. The recruitment threshold < 2% MVC-EMG level was defined as low-threshold motor units, 2%–4% as medium and > 4% as high. MUAP amplitudes of < 50 μV were defined as low-amplitude motor units, 50 μV–100 μV as medium and > 100 μV as high. The mean firing rate of < 8 pps was defined as low, 8 pps–14 pps as medium and > 14 pps as high. The residual analysis was performed to test whether the distribution of motor units with each firing property (e.g., high-threshold motor units) differed between the two motor tasks.

Results were presented as either mean (SD) or median (first, third quartiles).

## 3 Results

### 3.1 Performance of the two motor tasks undertaken

The peak isometric knee extension torques during MVC measurements before and after the two motor tasks were 118.2 (25.5) and 119.5 (26.4) Nm, respectively, and were not significantly different (*p* = 0.54). The average muscle activity during the plateau phase while performing the trapezoidal contraction was 14.4 (0.7)% MVC-EMG level for the voluntary task and 14.3 (0.8)% MVC-EMG level for the postural task, with no significant difference (*p* = 0.78). The simulated EMG area between the postural and voluntary tasks was not significantly different [0.18 (0.14, 0.28) mVs, 0.15 (0.12, 0.25) mVs, respectively; *p* = 0.59]. The force level during the plateau phase (15% MVC-EMG level) in the voluntary task corresponded to 37.5 (10.7)% MVC force.

### 3.2 Average data of the motor unit firing properties for each individual participant

**FIGURE 3:**
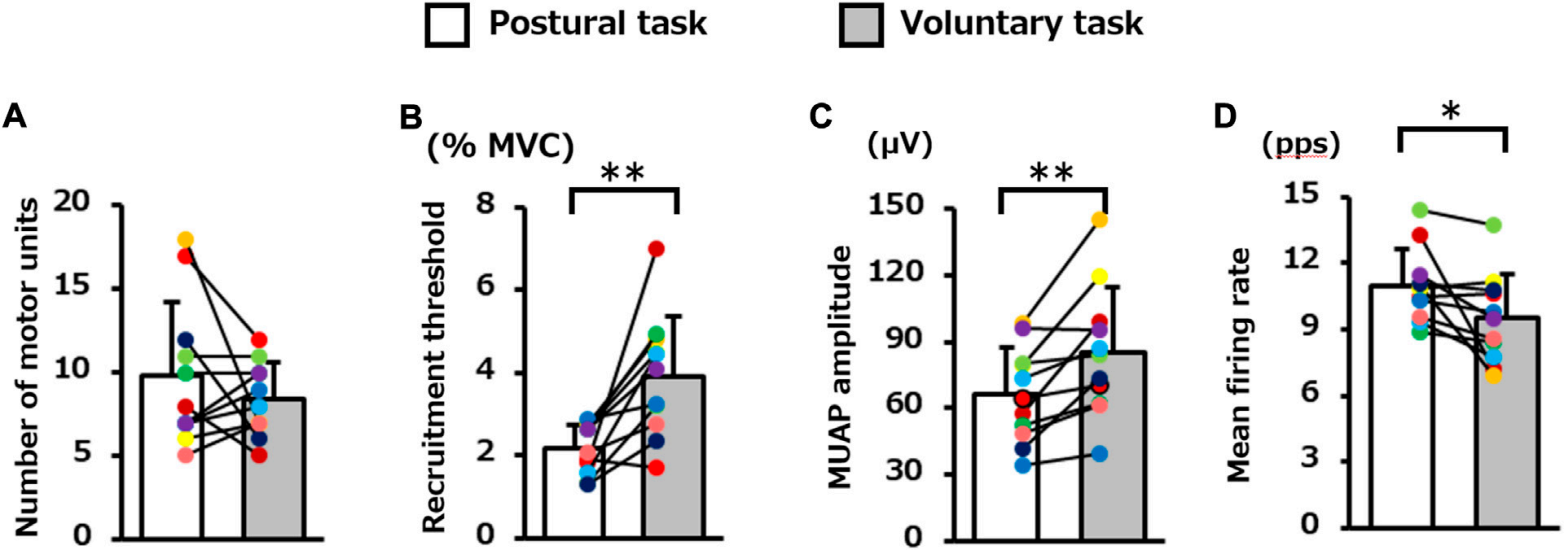
Difference in the motor unit firing properties between the two motor tasks. The bar graph presents; the **(A)** number of motor units, **(B)** recruitment threshold, **(C)** motor unit action potential (MUAP) amplitude, and **(D)** mean firing rate during the plateau phase in the postural and voluntary tasks (Paired paired *t*-test). Each color represents the average value obtained from each participant. **p < 0.01, *p < 0.05.

The average number of motor units identified per participant in the voluntary and postural tasks was 8.4 (2.2) and 9.8 (4.4), respectively, and these did not differ significantly (*p* = 0.29, Figure 3A). The decomposition accuracy of these motor units was 88.2 (1.1)% for the postural task and 90.7 (1.7)% for the voluntary task. The recruitment threshold was significantly higher for the voluntary task (3.9 [1.5]% MVC-EMG level) than for the postural task (2.2 [0.6]% MVC-EMG level) (*p* = 0.002, Figure 3B). MUAP amplitude was significantly lower in the postural task (66.2 [21.3] μV) than in the voluntary task (85.3 [29.2] μV) (*p* = 0.004, Figure 3C). The mean firing rate was 9.5 (2.0) pps in the voluntary task and 11.0 (1.7) pps in the postural task and was significantly higher in the postural task (*p* = 0.03, Figure 3D).

### 3.3 Relationship between recruitment threshold and mean firing rate

**FIGURE 4:**
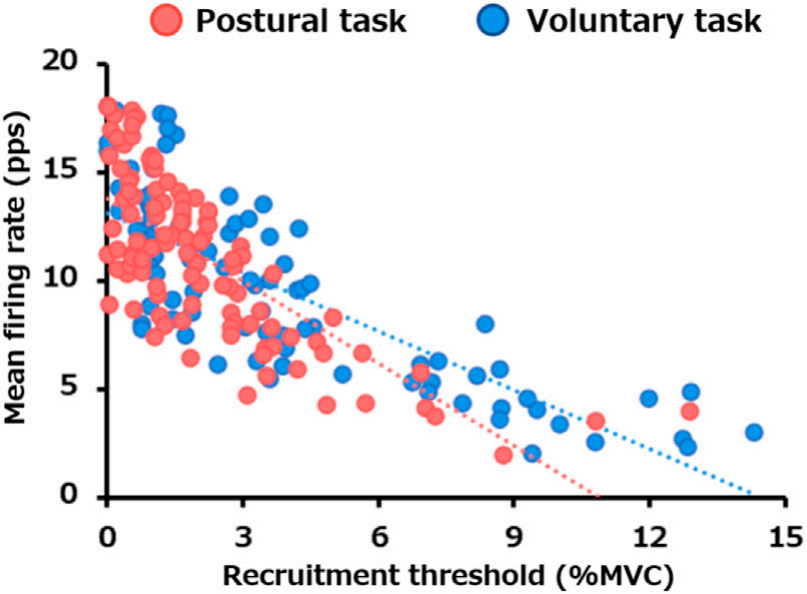
Difference in the relationship between the recruitment threshold and mean firing rate between the two motor tasks. The scatter plot presents the relationship between the recruitment thresholds and mean firing rates for all motor units pooled from all participants in the postural (red) and voluntary (blue) tasks.

The linear regression between the recruitment threshold and mean firing rate is presented in Figure 4. There were significant negative correlations between the motor unit recruitment thresholds and mean firing rate for both postural (*p* < 0.0005) and voluntary tasks (*p* < 0.0005). The slope of the regression line was −0.90 for the voluntary task (*R* ^2^ = 0.63) and −1.27 for the postural task (*R* ^2^ = 0.55), with the latter being significantly steeper (*p* = 0.007).

### 3.4 Firing properties of the motor units recruited in both motor tasks

**FIGURE 5:**
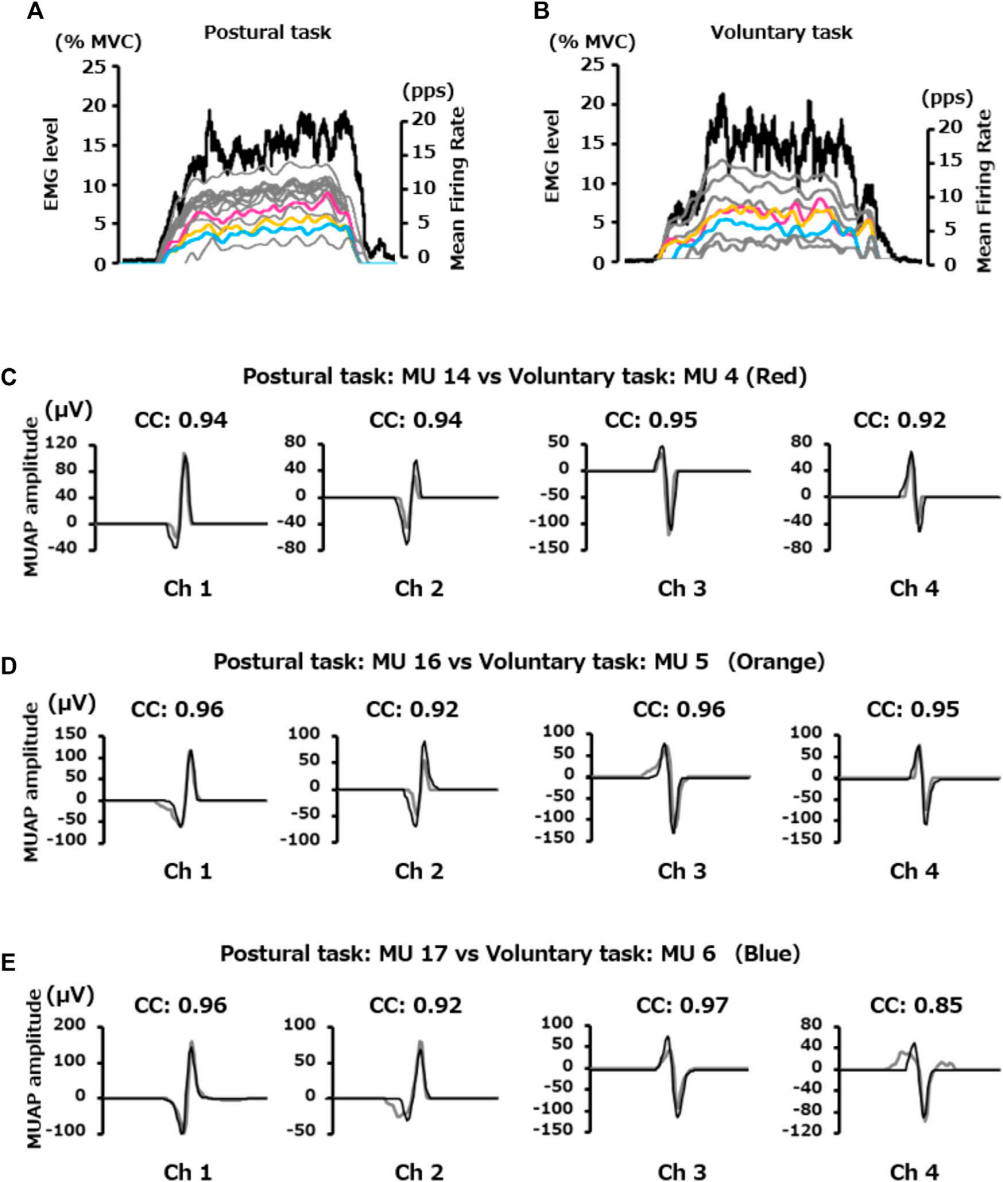
Difference in the firing properties of the motor units identified as identical between the two motor tasks. The upper two figures show the EMG level (black line) exerted in **(A)** postural and **(B)** voluntary tasks and the change in the firing rate of each motor unit. Red, orange, and blue lines indicate motor units shared by the two motor tasks, while the grey lines indicate motor units extracted independently from each motor task. Three pairs of motor units were identified as the same ones between two motor tasks **(C–E)**. The black and grey lines indicate the shape of the motor units in the postural and voluntary tasks, respectively. The values represent the cross correlation (CC) of the shape of the motor units in the two motor tasks.

**FIGURE 6:**
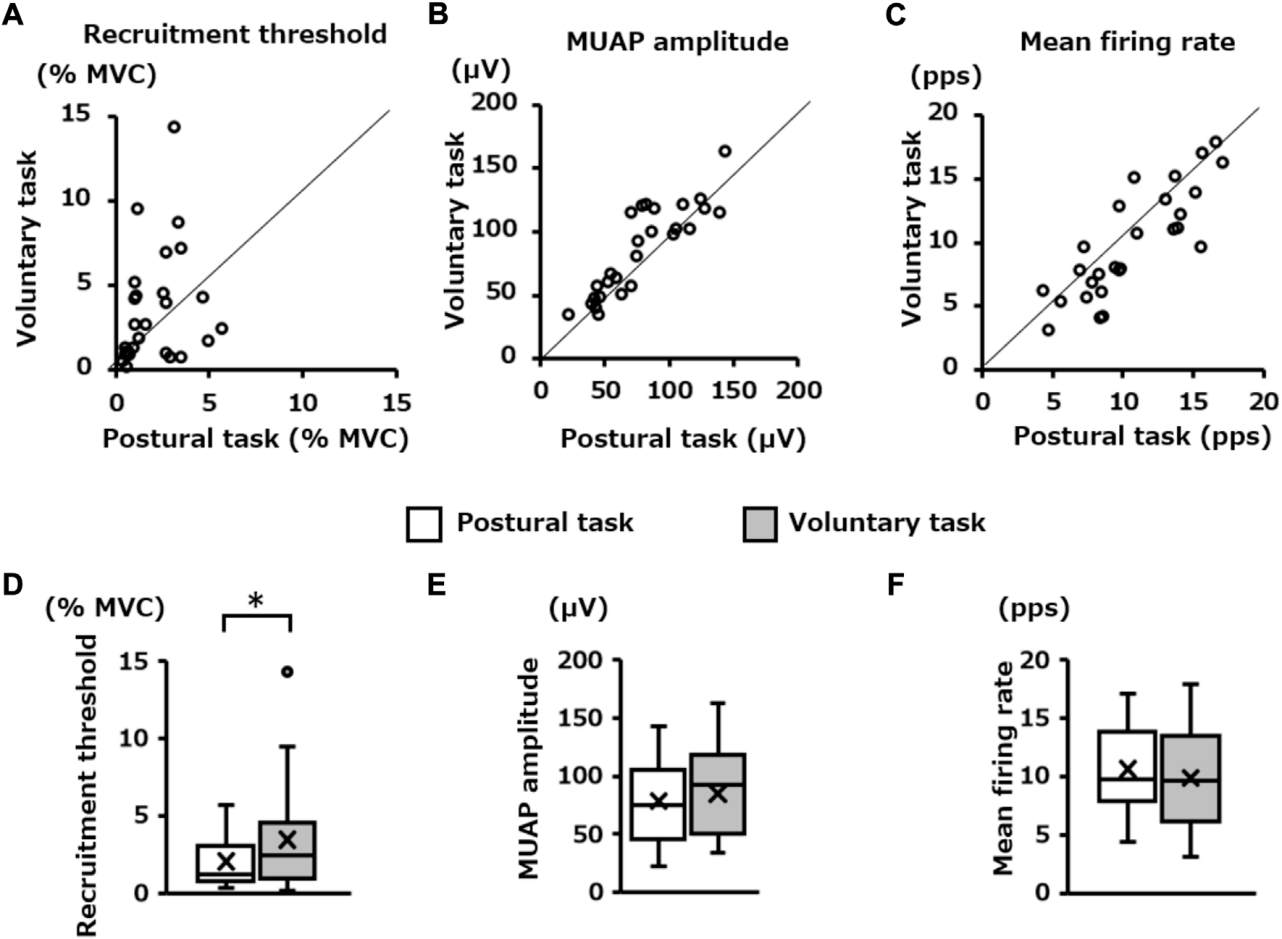
Difference in the firing properties of the same motor units between the two motor tasks. The scatter plots in the upper panel show the relationship in; **(A)** recruitment threshold, **(B)** motor unit action potential (MUAP) amplitude, and **(C)** mean firing rate of the pooled motor units that were determined to be identical between the postural and voluntary tasks. The box plots in the lower panel **(D,E,F)** show their statistical results (Wilcoxon Signed-rank Test) for each of them. *p < 0.05.

The number of motor units pooled from all participants was 108 for the postural task and 92 for the voluntary task. Among them, the number of motor units identified as common in the two motor tasks was 27 pairs (Figure 5). The scatter plots show the relationship in the firing properties (recruitment threshold, MUAP amplitude, and mean firing rate) of those 27 pairs of motor units between the two motor tasks (Figures 6A–C). Wilcoxon’s signed-rank sum test showed that the recruitment threshold of these motor units was significantly lower for the postural task (1.2 [0.8, 3.1]% MVC-EMG level) than for the voluntary task (2.4 [1.0, 4.5]% MVC-EMG level) (*p* = 0.02, Figure 6D). The MUAP amplitudes were 75.0 (46.1, 105.6) μV and 92.4 (50.5, 118.1) μV in the postural and voluntary tasks, respectively and these did not differ significantly (*p* = 0.10, Figure 6E). The mean firing rate of the motor unit was 9.6 (6.2, 13.4) pps in the voluntary task and 9.8 (7.8, 13.9) pps in the postural task, and there was no significant difference between them (*p* = 0.11, Figure 6F).

### 3.5 Firing properties of motor units recruited only in one of the motor tasks

**FIGURE 7:**
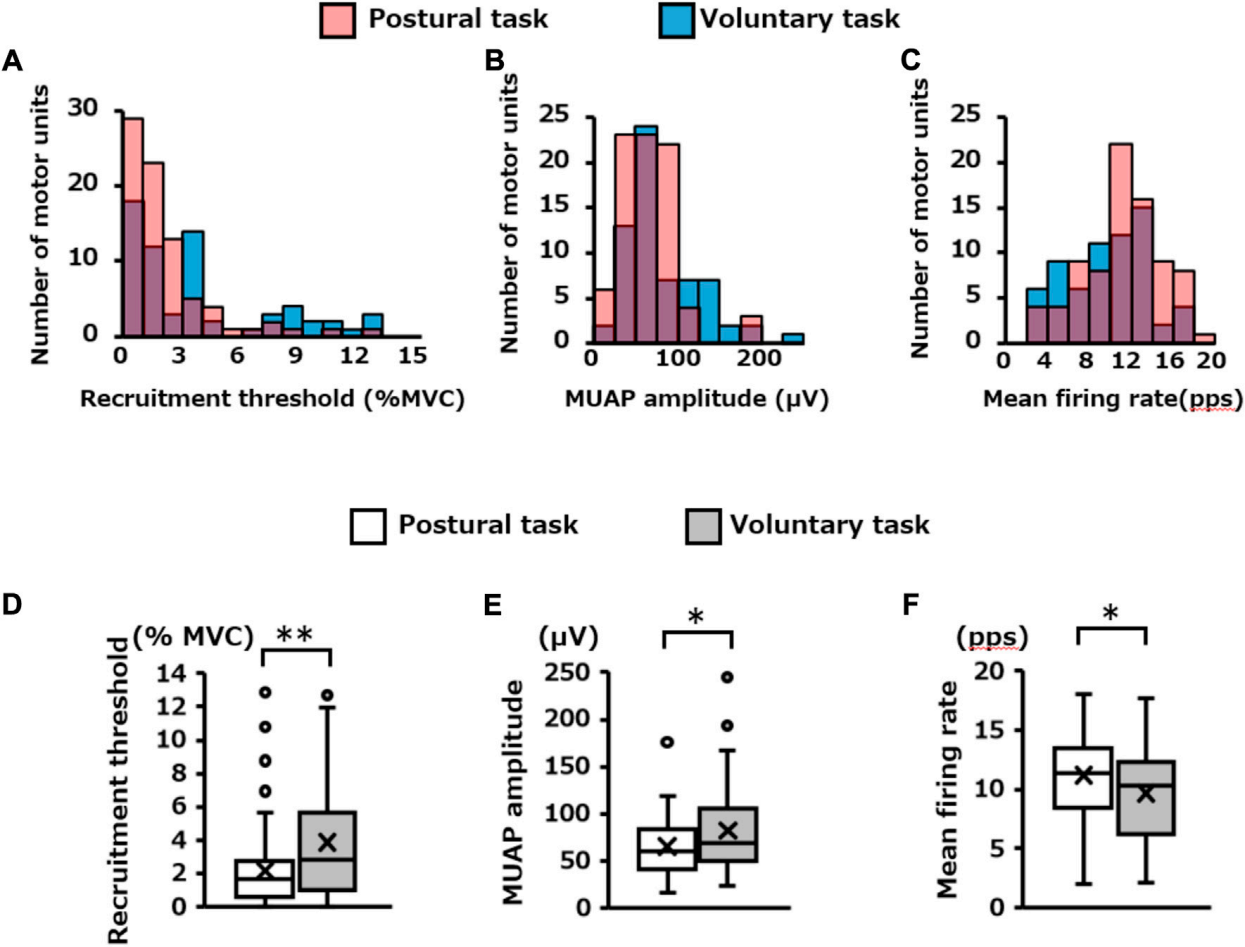
Firing properties of independently extracted motor units for each motor task. The histogram in the upper panel shows the distribution of the **(A)** recruitment threshold, **(B)** MUAP amplitude, and **(C)** mean firing rate for the pooled motor units extracted independently in each motor task. The red and blue bars indicate the results of the postural and voluntary tasks, respectively, and the purple areas indicate the overlap between the two. The box plots at the bottom show the statistical results (Mann–Whitney *U* Test) for each of them **(D,E,F)**. *p < 0.05, **p < 0.01.

The number of motor units extracted specifically for each motor task was 81 for the postural task and 65 for the voluntary task. The distribution of these motor units in each recruitment threshold, MUAP amplitude, and mean firing rate are presented in Figures 7A–C, respectively. Mann–Whitney *U* test revealed that the recruitment threshold and MUAP amplitude were significantly lower in the postural task (1.7 [0.6, 2.8]% MVC-EMG level, 60.3 [41.1, 83.0 μV)]) than in the voluntary task (2.9 [1.0, 5.6]% MVC-EMG level, 69.3 [50.4, 105.8] μV) (recruitment threshold; *p* = 0.003, Figure 7D, MUAP amplitude; *p* = 0.03, Figure 7E). In contrast, the mean firing rate was significantly higher in the postural task (11.4 [8.4, 13.5] pps) than in the voluntary task [10.4 (6.2, 12.4) pps] (*p* = 0.02, Figure 7F). Residual analysis revealed that the frequency of low-threshold motor units (< 2% MVC-EMG level) was significantly higher in the postural task (*p* = 0.03), whereas the frequency of high-threshold motor units (> 4% MVC-EMG level) was significantly lower (*p* = 0.03, Table 1). High-amplitude motor units (> 100 μV) were significantly less frequent in the postural than in voluntary task (*p* = 0.001). The frequency of motor units with a high firing rate (> 14 pps) was significantly higher in the postural compared to voluntary task (*p* = 0.04).

**TABLE 1:** Distribution of independently extracted motor units based on their firing properties.

		Postural task		Voluntary task	
		*n* (%)	ASR	*n* (%)	ASR
Recruitment threshold	Low (< 2% MVC)	52 (64.2)	2.18*	30 (46.2)	−2.18*
	Medium (2%–4% MVC)	18 (22.2)	−0.55	17 (26.2)	0.55
	High (> 4% MVC)	11 (13.6)	−2.12*	18 (27.7)	2.12*
MUAP amplitude	Low (< 50 μV)	29 (35.8)	1.67	15 (23.1)	−1.67
	Medium (50–100 μV)	45 (55.6)	0.95	31 (47.7)	−0.95
	High (> 100 μV)	7 (8.6)	−3.23**	19 (29.2)	3.23**
Mean firing rate	Low (< 8 pps)	17 (21)	−1.55	21 (32.3)	1.55
	Medium (8–14 pps)	46 (56.8)	−0.20	38 (58.5)	0.2
	High (> 14 pps)	18 (22.2)	2.10*	6 (9.2)	−2.10 *

ASR, adjusted standardized residual. *p < 0.05, ** *p*< 0.01

## 4 Discussion

To the best of our knowledge, this study is the first to investigate whether the motor units firing properties of the vastus lateralis muscle differ between the postural and voluntary tasks with both muscle activity levels matched. As a result, the recruitment threshold and MUAP amplitude were significantly lower in the postural task than in the voluntary task, and conversely, the mean firing rate was significantly higher. These results suggest that the firing properties of motor units clearly differ between postural and voluntary muscle contractions.

### 4.1 Differences in motor unit firing properties between two motor tasks

The order of recruitment of the motor units follows Henneman’s size principle (Henneman, 1957). However, factors other than the size of the motor units can affect their firing properties. For instance, Kennedy and Cresswell (2001) compared the motor unit recruitment threshold of the gastrocnemius muscle in knee flexion and extension position, in which the results showed that when the gastrocnemius muscle was shortened in the knee flexion position, the recruitment threshold was significantly higher. Therefore, the firing properties of the motor units are influenced by the muscles’ biomechanical factors. Additionally, the recruitment and the rate coding of motor units also changed depending on the motor task. In an isometric contraction, the recruitment order based on the size principle is maintained; however, in dynamic repeated movement, the order is reported to be disrupted (Thomas et al., 1987). Several previous studies showed that the order of recruitment of motor units of the FDI changed depending on the direction of the thumb or the index finger movement (Desnedt and Gidaux, 1981; Thomas et al., 1986). Furthermore, in the biceps brachi muscle, which is involved in elbow flexion and forearm supination, some motor units were selectively recruited only during either movement (ter Haar Romeny et al., 1982; van Zuylen et al., 1988). Hudson et al. (2017) showed that the motor units of the intercostal muscles in the first intercostal space were recruited before those of the fourth intercostal space during resting breathing, whereas the order was reversed during trunk rotation movement. Therefore, evidently the recruitment of motor units and their order are both influenced by the motor task. In this context, Loeb. (1985) referred to the group of motor units activated for a particular task as a “task groupˮ. The present results on the differences in the motor unit firing properties between the two motor tasks are consistent with the idea of this “task groupˮ.

Few studies have investigated the differences in firing properties of motor units between postural and voluntary muscle contraction. Mochizuki et al. (2006) studied the firing properties of single motor units from the soleus muscle during postural and voluntary tasks. The results showed that the discharge rate of the soleus was higher during the postural compared to the voluntary task, which partially supports the results of the present study. However, in their study, the muscle activity levels were not unified in the two motor tasks. In fact, EMG activity in the postural task was 43% higher than in the voluntary task. Since there was a linear relationship between the amount of muscle activity and the firing rate of motor units (Casolo et al., 2021), it is unclear whether the higher firing rate in the postural task was due to the differences in the motor task or muscle activity level. Another study compared the motor units firing properties in quadriceps femoris muscles between the voluntary knee extension task (open kinetic chain) and leg press task [closed kinetic chain (CKC)] (Boccia et al., 2019). As a result, the mean firing rate and the recruitment threshold at high torque levels (> 50% MVC) were both significantly higher in the knee extension task than in the leg press task. The voluntary task in the sitting position used in our study was consistent with the knee extension task in their study. In contrast, although the postural task used in our study corresponds to the CKC movement as well as the leg press task used in their study, there are some differences; our study used a postural task, whereas the other study used a CKC task in the supine position, in which a custom-built board connected to a dynamometer was pushed horizontally. Therefore, the results of our study cannot be simply compared with those of their study. Furthermore, in their study, similar to the aforementioned study reported by Mochizuki et al. (2006) the muscle activity levels were inconsistent between the two motor tasks, showing significantly higher muscle activity levels in the voluntary knee extension task compared to leg press task at force levels exceeding 50% MVC. This result may explain why the knee extension task had a significantly higher recruitment threshold and mean firing rate than the leg press task when exerting force above 50% MVC. Therefore, the current study was the first to show that motor unit firing properties were different between voluntary and postural tasks, even though they had the same muscle activity level, which provides important implications for training and rehabilitation using these motor tasks.

To test whether the firing properties of the same motor units changed with the motor task or whether distinct motor units with different properties were independently recruited in the two tasks, we identified motor units that are commonly recruited in both postural and voluntary tasks. As a result, the recruitment threshold of the shared motor unit was significantly lower in the postural task than in the voluntary task. Previous studies have shown that changes in recruitment thresholds for the same motor unit are also caused by changes in muscle shortening and lengthening velocity (Oliveira and Negro, 2021). Alternatively, there was no significant difference in the MUAP amplitude and mean firing rate between the two motor tasks in the shared motor units. For independently identified motor units in the two motor tasks, recruitment threshold and MUAP amplitude were significantly lower, and the mean firing rate was significantly higher in the postural task than in the voluntary task. Furthermore, these differences were attributed to the fact that the postural task had a lower percentage of high threshold (> 4% MVC-EMG level) and high-amplitude (> 100 μV) motor units than the voluntary task, and conversely, a higher percentage of low threshold (< 2% MVC-EMG level) and high firing rate (> 14 pps) motor units. These results suggest that the differences in the motor unit firing patterns in the two motor tasks are caused by differences in the nature of the independently recruited motor units, rather than by changes in the rate coding of the motor units common to the two tasks. In general, postural muscles are composed of a high proportion of fatigue-resistant S-type motor units (Burke et al., 1974) because of the need for sustained muscle contraction. Therefore, our findings that relatively low-threshold motor units are preferentially recruited in the postural task would be reasonable considering the resistance of motor units to fatigue.

### 4.2 Neurophysiological difference between postural and voluntary tasks

Several previous studies have shown that there is a negative correlation between recruitment threshold and motor unit firing rate, implying that low-threshold motor units tend to have high firing rates, whereas high-threshold ones tend to have low firing rates (Sterczala et al., 2020; Reece and Herda, 2021). The slope of correlation between the recruitment threshold and firing rate is considered to be an indicator of neural drive to the muscle (Reece and Herda, 2021). In the present study, we found a strong negative correlation between the recruitment threshold and mean firing rate in both the motor tasks. Furthermore, the slope of this correlation was significantly steeper in the postural than in the voluntary task. Thus, these results suggest that the vastus lateralis muscle may receive different neural drive between the postural and voluntary tasks, as described below.

The most important neurophysiological mechanism explaining the differences in the firing properties of motor units between the two motor tasks is the alterations in the descending input to the spinal motoneurons. Voluntary muscle contraction mainly involves the corticospinal and rubrospinal tracts (Heckman and Enoka, 2004). These neural pathways inhibit low-threshold motor units and predominantly activate high-threshold motor units (Burke et al., 1970; Endo et al., 1975; Powers et al., 1993) which supports our finding of the high-threshold motor units being more likely to be recruited in voluntary tasks than in postural tasks. In contrast, the reticulospinal tract and vestibulospinal tract are considered mainly responsible for the postural muscle contraction, which require automatic postural adjustment (Heckman and Enoka, 2004). In particular, it was shown that the reticulospinal system contains fibers that release serotonin and noradrenaline to spinal motoneurons, contributing to the regulation of the baseline excitability of spinal motoneurons (Jacobs et al., 2002). These descending monoaminergic drives to spinal motoneurons are considered to contribute to the sustained activation of low-threshold motor units at low synaptic inputs by further reinforcing their tendency to have the lowest threshold for recruitment (Lee and Heckman, 1998). Therefore, the recruitment threshold, which was significantly lower in the postural task might be due to differences in descending projection on the spinal motoneurons between the two motor tasks.

The second relevant factor associated with the difference in motor unit firing properties between the two motor tasks is the presence or absence of cutaneous afferent input from the sole of the foot, as it contacts the floor surface in the postural task and does not in the voluntary task. Nakajima et al. (2006) demonstrated that the modulation of cutaneous reflexes in the lower leg muscles induced by cutaneous stimulation of the sole of the foot was different between the sitting and standing positions. Therefore, the influence of sensory afferent input from the sole of the foot on muscle activity possibly differs depending on the posture of sitting and standing. Furthermore, the stimulation of the cutaneous afferent from the foot indicated different effects depending on the type of motor unit of the gastrocnemius muscle (Burke et al., 1970). Although it has not been clarified how the cutaneous sensory input from the sole of the foot affects the motor unit activity in the vastus lateralis muscle, the difference in sensory input from the sole of the foot between the postural and voluntary tasks could explain the difference in the motor unit firing properties between the two tasks.

The postural task in the standing position, compared with the voluntary task in the sitting position, requires the activity of the antagonistic hamstring muscles (Graham et al., 1993). Therefore, it is necessary to consider how the synaptic input from the muscle spindle afferents that is associated with muscle contraction of the hamstring affects the differences in the motor unit firing properties of the vastus lateralis muscle. In this context, it has long been shown that electrical stimulation of Ia fibers produces disynaptic reciprocal inhibition of the antagonist muscles (Hultborn, 2006). Burke and Rymer. (1976) reported that the amount of inhibitory synaptic input to motoneurons innervating antagonist muscles produced by electrical stimulation of Ia fibers from cat ankle flexors was greater in type S motor units. Moreover, Aimonetti et al. (2000) investigated the effect of Ia reciprocal inhibition produced by conditioned stimulation of the human median nerve on the motor unit firing probability of the hand extensor muscle. Consequently, the firing probability of the wrist extensor muscle reduced in low-threshold motor units, supporting the results of Burke and Rymer. (1976). These results are contradictory to those of the present study, which showed that the low-threshold motor unit was more likely to be recruited in the postural task than in the voluntary task. Therefore, the difference in Ia input due to the presence or absence of antagonist muscle contraction cannot explain the results of this study.

### 4.3 Methodological considerations and limitations

In this study, the MUAP amplitude, which is one of the firing properties of the motor unit, was compared between the two motor tasks. However, the distance between the recording electrodes and motor unit affects the MUAP amplitude (Roeleveld et al., 1997b; a; Rodriguez-Falces et al., 2013). Therefore, this might be partly associated with the difference in the MUAP amplitude observed between the two motor tasks.

In the postural task, it is impossible to make the participants exert the same force as in the voluntary task because the knee extension force level cannot be measured accurately. Therefore, in this study, EMG-RMS was used as feedback to the participants (Westgaard and De Luca, 2001; De Luca et al., 2006). Although EMG-RMS was equivalent between the two tasks, the possibility that actual muscle activity was different in the two motor tasks cannot be completely ruled out because the magnitude of EMG phase cancellation is affected by the number of recruited motor units and their firing properties (Keenan et al., 2005; Keenan et al., 2006). However, the simulated EMG area calculated from all extracted MUAPs and their numbers of firings did not significantly differ between the two motor tasks, suggesting that the effect of EMG amplitude cancellation was relatively small.

Since the results of our preliminary experiments showed that the postural task often had difficulty in producing a strong EMG level > 15% MVC, only those motor tasks with a 15% MVC-EMG level were included in this study. As a result, 15% MVC-EMG level was comparable to 37.5% MVC force levels in the voluntary task. This discrepancy between EMG and force levels in the vastus lateralis muscle is consistent with the results of a previous study (Saito and Akima, 2013). Some previous studies that investigated the behavior of motor units in the vastus lateralis used 30%–80% MVC as the target force level (Boccia et al., 2019; Mota et al., 2020; Reece and Herda, 2021). Additionally, new motor units were recruited even > 70% MVC force level in the vastus lateralis muscle (De Luca and Hostage, 2010). Therefore, the present results should be interpreted with caution, as we did not capture the behavior of motor units with a high recruitment threshold. Therefore, further investigation is warranted.

## 5 Conclusion

This study investigated whether the firing properties of the motor units of the vastus lateralis differ between the postural task and voluntary task. We showed that the mean firing rate was significantly higher in the postural task than in the voluntary task, and conversely, the recruitment threshold and MUAP amplitude were significantly lower. These differences were attributed to the fact that the postural task had a lower percentage of high threshold and high-amplitude motor units than the voluntary task, and conversely, a higher percentage of low threshold and high firing rate motor units. Preferential activation of fatigue-resistant motor units in the postural task is a reasonable strategy as it allows for sustained postural maintenance. These findings may have implications as a theoretical basis for choosing either the postural or the voluntary task in rehabilitation and sports training.

## Data Availability

The original contributions presented in the study are included in the article, further inquiries can be directed to the corresponding author.
